# Molecular detection of *Chlamydia psittaci* in wild birds, broiler chickens, and sheep in Egypt: a public health concern

**DOI:** 10.1186/s12917-026-05414-x

**Published:** 2026-04-06

**Authors:** Dalia A. Hamza, Rahma M. Rabiee

**Affiliations:** Department of Zoonoses, Faculty of Veterinary Medicine, Cairo University, PO Box 12211, Giza, Egypt

**Keywords:** *Chlamydia psittaci*, Chicken, Wild bird, Sheep, PCR, 16S rRNA, *ompA*, Egypt

## Abstract

**Background:**

*Chlamydia psittaci* is a neglected pathogen of global concern, characterized by significant zoonotic and spillover potential, posing considerable risks to public health. This study aimed to investigate the prevalence of *C. psittaci* in broiler chickens, wild resident birds, and sheep in Egypt using molecular detection and sequence analysis to explore possible zoonotic relevance.

**Methods:**

A total of 230 oropharyngeal swabs were collected from apparently healthy animals, including broiler chickens (*n* = 100), resident wild birds (*n* = 80; hooded crows *n* = 20, cattle egrets *n* = 20, rock pigeons *n* = 20, and laughing doves *n* = 20), and sheep (*n* = 50). Genomic DNA was extracted and screened using PCR targeting the *Chlamydia* genus-specific 16S rRNA   gene. Species identification of *C. psittaci* was confirmed by amplification of the *ompA* gene. Moreover, sequencing and phylogenetic analysis of the *ompA* gene were performed.

**Results:**

The overall prevalence of *C. psittaci* was 3.5% (8/230). Positive samples were identified in broiler chickens (5/100; 5%), hooded crows (1/20; 5%), rock pigeons (1/20; 5%), and sheep (1/50; 2%), while cattle egrets and laughing doves tested negative. Phylogenetic analysis of the *ompA* gene sequence showed close clustering with previously reported *C. psittaci* strains from seabirds, confirming a strong genetic relationship.

**Conclusion:**

The detection of *C. psittaci* in broiler chicken, wild birds, and sheep within the same geographic area highlights the potential circulation of this pathogen among multiple host species. These findings emphasize the importance of developing targeted surveillance systems and implementing One Health control strategies.

## Introduction

Avian chlamydiosis has emerged as an important global public health concern, threatening both animal and human health across multiple continents [1]. The disease affects a wide range of hosts, including humans, domestic animals, and wildlife, and its capacity for cross-species transmission underscores its zoonotic potential [2, 3]. The primary causative agent, *Chlamydia psittaci* (formerly *Chlamydophila psittaci*), is a Gram-negative, obligate intracellular bacterium belonging to the family Chlamydiaceae. Several other chlamydial species, including *C. avium*, *C. gallinacea*, *C. abortus*, *C. pecorum*, and *C. trachomatis*, have also been implicated in avian and mammalian infections [4, 5].

*Chlamydia psittaci* is a globally recognized zoonotic pathogen capable of infecting more than 500 avian species ranging from poultry to companion and wild birds [6–9]. Beyond avian hosts, the bacterium has also been detected in several mammals, including cattle, sheep, and horses, demonstrating its broad host range and the complexity of interspecies transmission cycles [10, 11]. Considerable genetic diversity has been reported among *C. psittaci* strains, with genotypes classified primarily based on outer membrane protein A (*ompA*) gene sequences. These genotypes often display host associations that contribute to differences in pathogenicity, transmission dynamics, and epidemiology across species [12].

The organism is shed in feces, respiratory and nasal secretions, placentas, and birth fluids of infected animals and is notably resistant to environmental desiccation, allowing it to survive for extended periods and increasing opportunities for exposure [13]. Humans and animals typically acquire infection through inhalation of contaminated aerosols or direct contact with infected birds, sheep, or their tissues. Additional transmission routes include bird bites, mouth-to-beak contact, and handling or consumption of contaminated poultry products, including eggs [14–16]. Areas where domestic birds, wild resident birds, and livestock share grazing fields or water sources are particularly vulnerable to pathogen circulation. Such ecological overlap facilitates contamination of shared environments with infectious particles through droppings, dust, feathers, and secretions, creating high-risk zoonotic interfaces [15]. These conditions pose a significant threat to farmers, veterinarians, poultry handlers, and others with occupational exposure, reinforcing the need for integrated One Health surveillance [10].

In humans, *C. psittaci* infection can result in clinical manifestations ranging from mild influenza-like illness to severe pneumonia and is responsible for approximately 1% of global community-acquired pneumonia (CAP) cases [17, 18]. In birds, the pathogen commonly induces respiratory, ocular, and gastrointestinal symptoms [10], whereas in sheep and other ruminants, *C. psittaci* has increasingly been recognized as an abortigenic agent [19, 20]. Numerous laboratory-confirmed outbreaks and sporadic cases of human psittacosis have been reported worldwide, linked to various avian species as well as sheep, highlighting the persistent zoonotic risk [21–25].

Despite extensive global documentation of avian chlamydial infections in domesticated birds [8, 26–28], data on pathogen circulation in wild birds and livestock, especially in regions where humans, poultry, and wildlife frequently interact, remain limited. This is particularly relevant in Egypt, where shared agricultural landscapes and close proximity between domestic and free-living birds may enhance cross-species transmission.

Therefore, this study aimed to investigate the prevalence of *Chlamydia psittaci* in broiler chickens, wild resident birds, and sheep in the same geographic area in Egypt using molecular detection methods, with preliminary sequence analysis to explore possible zoonotic relevance.

## Materials and methods

### Specimen collection

A total of 230 oropharyngeal swabs were collected from randomly selected, apparently healthy animals. These included 100 broiler chickens, 80 resident wild birds (20 hooded crows, 20 cattle egrets, 20 rock pigeons, and 20 laughing doves), and 50 sheep. Sampling was conducted between December 2024 and June 2025. Chicken samples were collected from multiple commercial farms in Giza Governorate, Egypt, operating under an intensive production system (deep litter system). Resident wild birds were free-living and humanely captured from farm-associated environments using modified traps; after swabbing, they were promptly released. Sheep samples were obtained from smallholder flocks.

All samples were immediately transported to the laboratory under sterile, refrigerated conditions to ensure sample integrity.

### DNA extraction

Genomic DNA was extracted using the DNeasy Blood & Tissue Kit (QIAGEN, Hilden, Germany) following the manufacturer’s guidelines. Extracted DNA was stored at − 20 °C until used in PCR amplification assays.

### Molecular detection of *Chlamydia* genus and *Chlamydia psittaci* by PCR

Uniplex PCR was performed to amplify the 16S rRNA gene using universal primers specific for the *Chlamydia* genus, following the method described by Everett et al. [29] and Borel et al. [30]. To confirm the presence of *C. psittaci*, the *ompA* gene, a species-specific marker, was targeted according to the method outlined by Heddema et al. [31]. The primer sequences and expected amplicon sizes are presented in Table 1.

**Table 1:** Oligonucleotide primer sets for PCR amplification of 16S rRNA and *ompA* genes for *Chlamydia psittaci* detection

Target gene	Primer sequence (5ʹ–3ʹ)	Amplicon size (bp)	Reference
*16S rRNA*	16 S-IGF: GAT GAG GCA TGC AAG TCG AAC G	278	[29, 30]
	16 S-IGR: CCA GTG TTG GCG GTC AAT CTC TC		
*OmpA*	CPsitt-F: GCTACGGGTTCCGCTCT	1041	[31]
	CPsitt-R: TTTGTTGATYTGAATCGAAGC		

Each PCR reaction was prepared in a total volume of 25 µL, consisting of 12.5 µL Emerald Amp MAX PCR Master Mix (Takara, Japan), 1 µL of each primer (10 pmol/µL; Metabion, Germany), 5 µL of template DNA, and PCR-grade water to adjust the volume.

The thermal cycling conditions for 16S rRNA gene were as follows: initial denaturation at 95 °C for 15 min; 45 cycles of denaturation at 94 °C for 30 s, annealing at 51 °C for 30 s, and extension at 72 °C for 45 s; followed by a final extension at 72 °C for 10 min. For, *ompA* the optimized conditions included an initial denaturation at 95 °C for 5 min; 30 cycles of denaturation at 94 °C for 1 min, annealing at 57 °C for 1 min, and extension at 72 °C for 1 min; with a final extension step at 72 °C for 7 min.

All PCR products were run on 1.5% agarose gel at 90 V for 45 min and visualized using a UV transilluminator. A 100 bp DNA ladder (range: 100–1000 bp; Jenna Bioscience GmbH, Jena, Germany) was run alongside the samples to determine the size of the PCR amplicons.

###  Sequencing and phylogenetic analysis

The PCR-amplified *ompA* gene fragment (1041 bp) obtained from a broiler chicken sample was purified using a QIAquick purification extraction kit (Qiagen, Hombrechtikon, Switzerland), following the manufacturer’s instructions. Purified PCR product was sequenced using the BigDye Terminator V3.1 sequencing kit (Applied Biosystems, Waltham, MA, USA), and the nucleotide sequence obtained was deposited in the National Center for Biotechnology Information (NCBI) GenBank database. The gene sequences were aligned against publicly available sequences in the NCBI GenBank database using the NCBI BLAST server (http://blast.ncbi.nlm.nih.gov/).Multiple sequence alignments and similarity analyses were calculated with the CLUSTALW 1.8^®^ program in BioEdit software (version 7.0.9.0). The Phylogenetic analysis was conducted with MEGA X software using the Maximum Likelihood method with 1000 bootstrap replicates.

## Results

### Prevalence of *Chlamydia psittaci* among examined host species

In this study, 230 oropharyngeal swab samples from avian and ovine hosts were screened by PCR, targeting the 16S rRNA gene for genus-level detection of *Chlamydia* and the *ompA* gene for species-level confirmation of *C. psittaci.* Overall, *C. psittaci* was detected in 3.5% (8/230) of the samples, with all eight positives amplifying both the 16S rRNA and *ompA* genes (Table 2).

**Table 2:** Prevalence of *Chlamydia psittaci* among avian and ovine hosts

Samples		Total number	Positive samples	
			*N*	%
Broiler chickens		100	5	5%
Wild resident bird	Hooded crow	20	1	5%
	Cattle egret	20	0	0%
	Rock pigeon	20	1	5%
	Laughing dove	20	0	0%
Sheep		50	1	2%
Total		230	8	3.5%

Across the sampled host species, *C. psittaci* was identified in 5 of 100 broiler chicken samples (5%), 1 of 20 hooded crow and 1 of 20 rock pigeon (each 5%), whereas cattle egret and laughing dove yielded no positive results. Ovine samples showed a prevalence of 2% (1/50) (Table 2).

### Molecular epidemiology and phylogenetic analysis of *ompA* sequences

The amplified *ompA* gene product obtained from a broiler chicken sample was subjected to DNA sequencing. The nucleotide sequence obtained was deposited in the National Center for Biotechnology Information (NCBI) GenBank database under the accession number (PX601580).

**Fig. 1:**
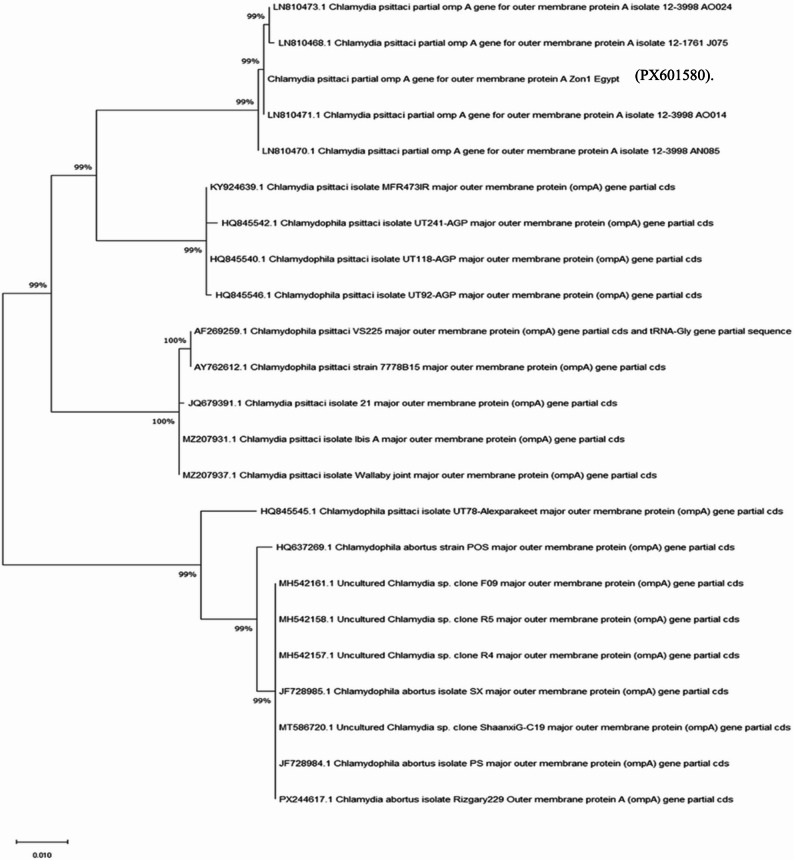
Comparison of FIV point-of-care kit test line intensity in two representative cats. For each panel, VDRG® kits (top) consistently showed weaker positive test lines compared with Fassisi® kits (bottom) when testing the same cat. Panel A (cat no. 218) illustrates the predominant VDRG® result pattern (36/38 positive samples) characterized by weak test lines, whereas Panel B (cat no. 104) represents one of the two VDRG®-positive cases with a more distinct test line. In both panels, Fassisi kits exhibit visibly stronger test lines

The phylogenetic tree shows the evolutionary relationship of the *ompA* gene sequences of *Chlamydia psittaci* and related Chlamydiaceae species. The sequence included in this study cluster strongly with previously published *C. psittaci* sequences, forming a well-supported monophyletic group with high bootstrap values (99–100%), indicating a close genetic relationship. Our sequence falls within the same clade as reference strains such as (LN810473, LN810468, and LN810471) (Fig. 1).

## Discussion

Avian chlamydiosis remains a global threat to animal health and the poultry industry, carrying major economic and trade implications [5, 20]. Moreover, the disease poses a significant public health concern, marked by elevated mortality rates [32]. However, epidemiological data on the distribution and prevalence of *C. psittaci* among avian and mammalian hosts in Egypt remain limited. In this study, an overall prevalence of 3.5% (8/230) was recorded in oropharyngeal swabs from broiler chickens, wild resident birds, and sheep, underscoring the organism’s broad host range and its potential for cross-species transmission.

Findings from chickens revealed that *C. psittaci* was detected in 5% (5/100) of samples, a figure comparable to the 6.9% prevalence reported in Slovakia [33], but markedly lower than the high rates observed in Belgium (85%) and Egypt (91.6%) [9, 34]. Such variances likely reflect differences in bird species, geography, sampling methods, and farm practices [8, 35]. In Egypt, the close confinement of multiple bird species in poultry farms and live bird markets generates considerable physiological stress, which promote bacterial shedding through respiratory secretions and feces [15, 36]. Consequently, leading to extensive environmental contamination, transforming these settings into key reservoirs that facilitate bird-to-bird transmission and increase the risk of zoonotic spillover to humans, consistent with previous findings [37].

Egypt’s heterogeneous habitats support diverse resident wild bird species, with cattle egrets, rock pigeons, hooded crows, and laughing doves being the most prevalent [38]. Among these species, *C. psittaci* was detected in hooded crows and rock pigeons, with a prevalence of 5% in each group. The ubiquity of these birds in urban and peri-urban settings, along with scavenging behaviors, heightens exposure to infected domestic birds and contaminated environments, thereby promoting pathogen acquisition and dissemination [39, 40]. In contrast, the absence of detectable infection in cattle egrets and laughing doves may reflect the intermittent shedding characteristic of *C. psittaci* [41]. Accordingly, broader surveillance efforts encompassing larger sample sizes and diverse ecological zones are necessary to better define the epidemiological roles of these wild species.

Although, *C. psittaci* is primarily known as an avian pathogen, its detection in oropharyngeal swabs from a clinically healthy sheep, with a prevalence of 2% (1/50), is noteworthy. This prevalence is lower than the 6.7% prevalence previously reported in fecal samples from sheep in Egypt by Osman et al. [11], a difference that may be explained by the variation in sample types, sampling strategy, and diagnostic methods used across the two studies. Such findings likely reflect the close ecological interactions inherent to Egypt’s mixed farming systems, where livestock often share grazing areas with domestic and wild birds. This proximity creates multiple pathways for transmission, including exposure to aerosols, droppings, feathers, respiratory secretions, and other contaminated shared resources [15]. These dynamics underscore the necessity of establishing comprehensive surveillance systems at the convergence of wildlife, domestic avian populations, and livestock.

Notably, detection of the bacterium in apparently healthy chickens, wild resident birds, and sheep underscores the largely subclinical nature of infection and supports the role of these hosts as covert reservoirs, capable of sustaining infection and facilitating silent transmission across species boundaries [42]. The epidemiological complexity of this pathogen presents major obstacles to the implementation of effective control and prevention strategies.

From a One Health perspective, these findings suggest potential public health implications, inferred from the detection of *C. psittaci* in animal reservoirs and supported by existing evidence of zoonotic transmission. Individuals with frequent occupational exposure to birds and livestock, including, farmers, veterinarians, poultry handlers, and abattoir worker, may face an elevated risk of psittacosis [10, 37]. Mitigating this risk requires coordinated surveillance systems, strengthened biosecurity practices, and cross-sector collaboration to protect public health [43].

To further elucidate the epidemiological context of circulating strains, phylogenetic analysis targeting the *ompA* gene was performed. This gene, which encodes the major outer membrane protein (MOMP), is widely regarded as a reliable and highly discriminatory molecular marker for *C. psittaci* detection and genotyping [44]. In the present study, one representative *ompA*-positive isolate from a broiler chicken was selected for sequencing and phylogenetic analysis. Although sequencing all positive samples would have strengthened the phylogenetic depth, the combined use of genus-specific 16S rRNA PCR and species-specific *ompA* amplification provides reliable molecular confirmation of *C. psittaci* in the examined hosts.

The constructed phylogenetic tree further revealed that the sequence obtained in this study clusters within a well-supported monophyletic group alongside reference strains derived from North Atlantic seabirds (LN810473, LN810468, and LN810471) [45]. This clustering was supported by high bootstrap values (99–100%), indicating strong statistical confidence, marked genetic conservation, and a shared evolutionary lineage. These molecular findings offer preliminary epidemiological insights reflecting the shared ecological interface among multiple hosts in Egypt. They also underscore the potential zoonotic relevance of the pathogen within interconnected avian populations and shared environments, thereby highlighting its public health significance.

Although the interpretation of host-specific prevalence is limited and epidemiological links were not directly evaluated, future studies incorporating larger sample sizes and multilocus or whole-genome sequencing are warranted to better characterize genetic diversity and transmission dynamics.

## Conclusion

The study sheds light on the prevalence and epidemiology of *C. psittaci* among diverse avian species and ovine hosts in Egypt. The findings reveal the role of these animals as reservoirs and highlight the potential for cross-species transmission within shared ecological settings. Collectively, these insights reinforce the urgent need for integrated surveillance under a One Health framework, along with strengthened biosecurity measures, to reduce transmission risks and protect both animal and human health.

## Data Availability

All the data generated or analyzed in this study are included in this published article.

## Abbreviations

CAP: Community-Acquired Pneumonia
DNA: Deoxyribonucleic Acid
Fig: Figure
MEGA: Molecular Evolutionary Genetics Analysis
MOMP: Major Outer Membrane Protein
NCBI: National Center for Biotechnology Information
OmpA: Outer Membrane Protein A gene
PCR: Polymerase Chain Reaction
rRNA: Ribosomal Ribonucleic Acid
UV: Ultraviolet
